# Ductile Effect of PGA/PCL Blending Plastics Using a Novel Ionic Chain Extender with Non-Covalent Bonds

**DOI:** 10.3390/polym15143025

**Published:** 2023-07-12

**Authors:** Hyuk-Jun Kwon, Joseph Jang, Won-Gun Koh, Jun-Young Lee, Kiseob Hwang

**Affiliations:** 1 Department of Chemical and Biomolecular Engineering, Yonsei University, Seoul 03722, Republic of Korea; hyukjun@kitech.re.kr (H.-J.K.); wongun@yonsei.ac.kr (W.-G.K.); 2 Green and Sustainable Materials R&D Department, Korea Institute of Industrial Technology, Cheonan 31056, Republic of Korea; jangy@kitech.re.kr

**Keywords:** ionic chain extender, non-covalent bonds, ductility, polyglycolic acid, blending plastics

## Abstract

Polyglycolic acid (PGA) is a promising polymer in the packaging field owing to its excellent hydrolysis, heat resistance, and gas barrier properties, but it is limited in application due to its poor toughness. For this reason, a covalently bonded chain extender is introduced to increase compatibility with flexible polymers. However, covalent bonds are unfavorable for application to degradable plastics because of the energy required for reverse reactions. Therefore, we intended to effectively control the ductility of blending plastics by using a novel ionic chain extender with a relatively weaker non-covalent bond than the existing covalent bond. Polycaprolactone (PCL), which has biodegradability and flexibility, was selected as a blending polymer. For comparison, a covalently reactive chain extender (G-CE) and a non-covalently ionic chain extender (D-CE) were synthesized and compounded with blending plastics. Each chain extender improved the compatibility between PGA and PCL, and the ductility of the PGA/PCL blending plastics was more greatly enhanced with non-covalently bonded D-CE than with covalently bonded G-CE. At this time, the ductility of the PGA/PCL(90/10) blending plastic without CE was 7.2%, the ductility of blending plastic with D-CE (10D) was 26.6%, and the ductility of blending plastic with G-CE (10G) was 18.6%. Therefore, it was confirmed that the novel ionic chain extender inducing non-covalent bonds improves the compatibility between PGA and PCL and is more advantageous in enhancing ductility through a reversible reaction.

## 1. Introduction

Polyglycolic acid (PGA) is composed of relatively simple aliphatic polyester and is one of the representative biodegradable plastics. PGA decomposes into smaller molecules by the hydrolysis of ester groups in the main chain and then finally decomposes into carbon dioxide through a biodegradation pathway in which the molecules are metabolized [1,2,3]. In addition, the chemical structure of PGA is simple aliphatic polyester without side chains. Therefore, the molecular structure has high density, high crystallinity, and a small free volume. For this reason, PGA shows a high level of mechanical strength compared to other biodegradable plastics [1,4,5]. Recently, an attempt was made to apply PGA in the packaging field because it became possible to produce PGA from synthesis gas and industrial tail gas [6,7].

However, it is limited in its application in the packaging field due to its brittleness and poor ductility, and some papers have reported that PGA has very low elongation at a break of less than about 5% [8,9]. In addition, there is difficulty in selecting the processing temperature because the pyrolysis temperature and melting temperature are about 250–280 °C and 220–230 °C, respectively [10,11]. Therefore, attempts have been made to improve the processability of PGA through the compounding of additives to increase the thermal decomposition temperature or through blending with a flexible polymer to improve ductility [12,13,14]. Recently, biodegradable polymers have been blended as flexible polymers such as poly(butylene adipate-co-terephthalate) (PBAT) [15,16,17], poly(propylene carbonate) (PPC) [18], polyhydroxyalkanoates (PHAs) [19], and polycaprolactone (PCL) [20]. However, most of the flexible polymers had a limited effect on improving the ductility due to low compatibility with a PGA matrix with strong inter-intermolecular interactions through hydrogen bonds [21,22]. Accordingly, chain extender (CE) as a compatibilizer was added to increase the interface interaction between the flexible polymer and PGA, and the most representative chain extender is Joncryl^®^ CE (BASF Inc., Florham Park, New York, USA). The Joncryl^®^ CEs have functional groups of epoxy or isocyanate and exhibit the characteristics of increasing viscoelasticity and reducing melt flow index through covalent bonds with the blending polymer chains [23,24,25].

In addition, these chain extenders improve the stretchability when used in an ap-propriate amount, but there is a problem in that the hardness increases due to a cross-linking reaction depending on the amount introduced, and the stretchability is inhibited. Many studies reported on the control of the ductility of PGA by using CEs, but all of the CEs were chemical-reactive compatibilizers due to covalent bonding [23,26]. These reactive CEs require a lot of energy for the reverse reaction, and they are hard to separate in the hydrolytic degradation process. Usually, the reactive CEs can inhibit the degradation rate due to the capping of the end group of PGA after covalent bonding. This behavior becomes a factor that hinders the use of hydrolyzable plastics [26,27].

Accordingly, we would like to propose that a new chain extender can control the ductility using an ionic bond with relatively weak bonding strength compared with a covalent bond. The ionic bonds are being applied in the field of self-healing because of repeated decomposition and recombination under conditions such as temperature or the presence of ions [28,29,30]. Some studies are reported where composites with quaternary ammonium (QA) can increase the fluidity of the polymer matrix and reduce frictional resistance under shear stress by causing a decrease in pour viscosity [31,32].

Therefore, a novel ionic chain extender was studied to control the ductility of brittle PGA and applied as a blending plastic to confirm the physical property behavior. Additionally, PCL was selected as a blending polymer owing to ionic bonding similar to PGA, which has a carboxyl group in the end group.

## 2. Materials and Methods

### 2.1. Materials

Methyl methacrylate (MMA), tert-butyl methacrylate (t-BMA), glycidyl methacrylate (GMA), and 2-(dimethylamino)ethyl methacrylate (DMAEMA) are monomers that were purchased from Sigma–Aldrich for chain extender synthesis. Furthermore, 2,2′-azobisisobutyronitrile (AIBN) as an initiator, toluene as a solvent, and hexane as a precipitation solvent after the synthesis of the chain extender, were also obtained from Sigma–Aldrich. The PGA (inherent viscosity 1.3–1.7 dL/g) was supplied by META BIOMED Co. Ltd., Republic of Korea, and the PCL (Mn 80,000 g/mol, Mw/Mn < 2) was obtained from Sigma–Aldrich. A Joncryl^®^ (ADR-4368), purchased from BASF Co. Ltd., was used to compare the mechanical properties of the blending plastics.

### 2.2. Preparation of Chain Extenders

The chain extenders were synthesized using different monomers according to the bonding method of the functional groups. The covalently bonded chain extender (G-CE) was synthesized by radical polymerization. The monomers (GMA 3 g, MMA 6 g, and t-BMA 6 g) were mixed with solvent (toluene 15 g) in round-bottom flasks with magnetic stirring at 200 rpm, and the mixture was thermostated in an oil bath at 70 °C in an argon atmosphere. Then, the initiator (AIBN 0.15 g, dissolved in 10 mL of solvent in advance) was slowly added dropwise for 1 h using a syringe pump and then synthesized for 4 h. After the reaction, the reactant was cooled to room temperature and washed, which was repeated 4 or more times by precipitation and re-dissolution using hexane. Finally, the product was obtained by drying overnight in a vacuum oven. The ionic chain extender (D-CE) was synthesized using the same method above, and was prepared by changing the monomers to DMAEMA 1.5 g, MMA 7.5 g, and t-BMA 6 g.

### 2.3. Compounding of PGA/PCL Blending Plastics with/without Chain Extenders

To confirm the effect of the synthesized chain extenders on the blending plastic, PGA and PCL were compounded according to their respective compositions as shown in Table 1. The PGA and PCL were dried overnight in a vacuum oven before compounding. The PGA/PCL blending plastics were prepared by an internal mixer (QM310S, QMESYS Inc., Uiwang-si, Republic of Korea) with a screw speed of 60 rpm at 230 °C. Then, the samples prepared through each compounding were formed into specimens at a temperature of 230 °C using a Hydraulic Laboratory Press (Carver Inc., Wabash, USA).

### 2.4. Characterization

The chemical structure of synthesized chain extenders was determined by proton nuclear magnetic resonance spectroscopy (^1^H-NMR, Bruker 500 MHz, Germany) with deuterated chloroform (CDCl_3_) as the solvent. The molecular weight of the synthesized chain extenders was measured using gel permeation chromatography (GPC, EcoSEC HLC-8420, Tokyo, Japan) under tetrahydrofuran (THF) solvent conditions. The melt flow index (MFI) of PGA/PCL blending plastics with/without chain extenders was measured using QM280A (QMESYS Inc., Uiwang-si, Republic of Korea) according to ASTM D1238, which measured at 230 °C and a loading of 2.16 kg. The mechanical properties of blending plastics were measured using a universal testing machine (UTM, QMESYS Inc., Uiwang-si, Republic of Korea) at a 10 mm/min crosshead speed. The MFI and UTM are measured 5 times per sample and provided as an average value. Different differential scanning calorimetry was analyzed to confirm the blending plastic’s crystallinity behavior according to the chain extenders’ introduction (DSC 8500, PerkinElmer Inc., Waltham, USA). Each sample was heated from 30 to 230 °C at 10 °C/min as first heating, held for 1 min at 230 °C, and cooled to −50 °C, before heating again to 250 °C at 10 °C/min while scanning as second heating. A thermogravimetric analysis (TGA) was used for the characterization of synthesized chain extenders. The condition of analysis was used at a temperature range of 30 °C to 600 °C under a high-purity nitrogen gas (99.99%) by TGA8000 model (TGA8000, PerkinElmer Inc., Waltham, USA). The morphologies of PGA/PCL blending plastics were obtained using a JSM-6701F field emission-scanning electron microscope (FE-SEM, JEOL Inc., Japan). Each PGA/PCL blending plastic sample was immersed in liquid nitrogen and then crushed, before being coated with gold for the cross-section measurements.

## 3. Results

### 3.1. Chemical Structure of the Chain Extenders which Functionalized into Different Groups

H-NMR analysis was conducted to confirm the chemical structures of the two synthesized chain extenders (Figure S1). The chain extenders were synthesized using similar monomers, whereas the functional monomer differed. The G-CE had epoxy functional groups capable of chemical bonding and the D-CE had tertiary amine functional groups capable of ionic bonding. Therefore, these chain extenders had a backbone similar to the methyl groups, indicating the peaks corresponding to polyacrylates derived from MMA and t-BMA. The typical peaks of methyl protons were measured and the signals of MMA, t-BMA, and backbone were observed at 3.5–3.7, 1.4–1.5, and 0.7–1.3 ppm, respectively (Figure S1a,b) [33].

On the other hand, these were confirmed to be different peaks of functional groups. The G-CE was shown to have epoxy groups as a functional group through the peaks at 2.6 and 2.8 ppm corresponding to -CH_2_ in the epoxy group (Figure S1a). And, the D-CE was confirmed by the peaks of tertiary amine groups at 2.3 ppm of -CH_3_ and 2.6 ppm of -CH_2_, which indicated that the tertiary amine functional groups capable of ionic bonding had been well introduced to the new chain extender (Figure S1b) [33,34]. The molecular weight analysis results for each synthesized chain extenders are provided in the supporting information (Table S1).

### 3.2. Evaluation of Changes in Melt Flow Index Characteristics according to Heat Exposure Times and Chain Extender Contents

**Figure 1:**
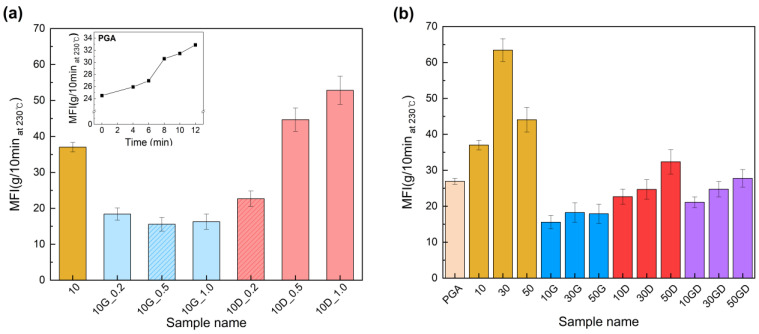
Melt flow index (MFI) of PGA/PCL/CE blending plastics according to ratio of chain extenders (**a**) and PGA/PCL blending with/without CEs according to blending ratio (**b**).

The melt flow index (MFI) was measured to determine the melt processability of PGA/PCL according to the blending ratio and the introduction of the chain extender (Table S2). In the first, the melt index of PGA over time was analyzed to explore the thermal decomposition time upon melting of PGA before the introduction of the chain extenders (Figure 1a, inside graph). The initial MFI value of PGA was 24.5 g/10 min, and the MFI increased as the heat treatment time increased. In particular, the MFI gradually increased to 26.94 g/10 min until the heat treatment time of 6 min, but rapidly increased to 30 g/10 min or more after 8 min, indicating that the thermal decomposition of PGA proceeded rapidly. Accordingly, the experiment was conducted by setting the PGA compounding time to within 6 min. And the thermal stability of synthesized CEs was confirmed by TGA analysis to set compounding temperature with PGA and PCL (Figure S2).

Furthermore, the MFI of the PGA/PCL blending plastics was analyzed, confirming the effect of introducing each synthesized chain extender (Figure 1a). When 0.2 wt% of the G-CE or D-CE was added, it was confirmed that the MFI significantly decreased compared to without the addition of any chain extenders. At this time, the MFI was measured at 18.4 g/10 min at 10G_0.2 and 22.7 g/10 min at 10D_0.2.

G-CE, with a covalent bond, did not essentially change its MFI according to the introduced amount of the chain extender. In detail, the lowest MFI was 15.6 g/10 min at 10G_0.5, and the MFI tended to increase slightly to 16.3 g/10 min at 10G_1.0. Since PGA and PCL have one carboxyl group at the end group, they can exhibit the highest molecular weight when fully reacted with the epoxy groups of the G-CE. However, when the chain extender is added in excess, the molecular weight is relatively reduced because the content of the epoxy groups is greater than the content of the adjacent carboxyl groups. As a result, G-CE tended to increase MFI relatively when added in amounts of 1 wt% or more.

On the other hand, the novel chain extender of D-CE, with an ionic bond, showed a high MFI compared to the control except for the content of 2 wt% (10D_0.2). The MFI measured in the PGA/PCL blending plastics according to the introduced amount of D-CE was 22.7, 44.7, and 52.8 g/10 min, corresponding to 10D_0.2, 10D_0.5, and 10D_1.0, respectively. Those ionic chain extenders form the ions from the quaternary ammonium groups for ionic bonding with the carboxylic groups of PGA. Moreover, the composite with quaternary ammonium (QA) can increase the fluidity of the matrix after mixing with polymer as PGA and PCL, and reduce the frictional resistance during shear stress by causing a decrease in pour viscosity [31,32]. For this reason, the MFI of the PGA/PCL blending plastics was significantly increased by using the D-CE with an ionic bond compared with introducing G-CE with a covalent bond. Here, we propose introducing reversible ionic chain extenders that dramatically change the melt viscosity of blending plastics over covalent bonds.

Next, the effects of the two chain extenders on the melt viscosity behavior of the PGA/PCL blended plastic were confirmed (Figure 1b). In general, as the content of PCL with a low melting point increases, the MFI of the PGA/PCL blending plastic increases. As shown in Figure 1b, the MFI of the sample according to the PCL ratio was 37.02, 63.42, and 44.07 g/10 min at 10, 30, and 50, respectively. Exceptionally, the ‘50’ sample, with a PCL content of 50%, showed a lower MFI than the ‘30′ sample. It can be inferred that the compatibility between the polymers is significantly lower than that of other samples based on the largest MFI error width for the sample. On the other hand, it was found that the samples to which the two chain extenders were added had significantly lower MFIs, and it was confirmed that the change in MFI according to the increase in PCL content was minor. As a result, it was confirmed that the two chain extenders improved the compatibility of PGA and PCL and induced low MFI through interaction with the polymer.

### 3.3. Crystallinity of PGA/PCL Blending Plastics according to Chain Extenders

**Figure 2:**
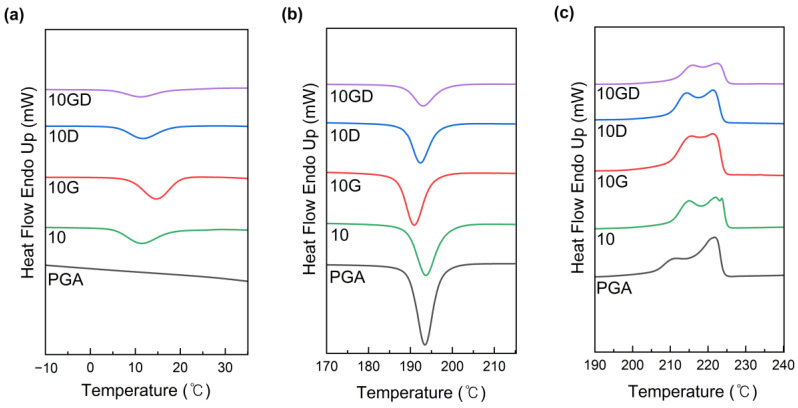
Differential scanning calorimetry (DSC) thermographs of neat PGA, PGA/PCL, and PGA/PCL/CEs blending plastic samples about Tc_1_ (**a**), Tc_2_ (**b**), and Tm (**c**).

DSC was analyzed for PGA/PCL blended plastics to confirm the effect of introducing covalent and non-covalent chain extenders on the crystallinity of polymers (Figure 2). In general, pure PGA has a chemical structure of high chain regularity without side chains, so it has a glass transition temperature (T_g_) of about 50 °C, a melting point ™ of about 200–225 °C, and a crystallization temperature (T_c_) of about 190–195 °C, which are higher than those of pseudolinear aliphatic polyesters [10,35]. These T_g_, T_m_, and T_c_ were almost unchanged when blended with PCL without a chain extender, suggesting that PCL had little effect on the thermal properties of PGA (Figure S2 and Table S3).

On the other hand, the crystallization temperature of PGA was shifted lower, and the interaction between PGA molecules was limited due to chain entanglement and cross-linking structure formation by covalent bonds.

Furthermore, the low T_c_ equivalent to T_c_ of PGA shifted to lower temperatures with the reaction with G-CE, suggesting more nucleation centers in the compatible system (Figure 2b). We reasoned that this was the effect of the nucleation of the domains of PGA or PCL and the nucleation impurities of their matrix polymers [35,36]. They inhibited the crystal growth process of entangled chains. Therefore, crystallinity, crystallization enthalpy, and melting temperature decreased. The PGA/PCL blend exhibited two melting endothermic peaks, suggesting that the crystal was not perfect due to the complexity of the macromolecular structure. G-CE shifted the crystallization temperature of PCL to a higher level and slightly increased the crystallinity of PCL segments due to covalent bonds.

### 3.4. Ductile properties of PGA/PCL Blending Plastics with Novel Chain Extenders

**Figure 3:**
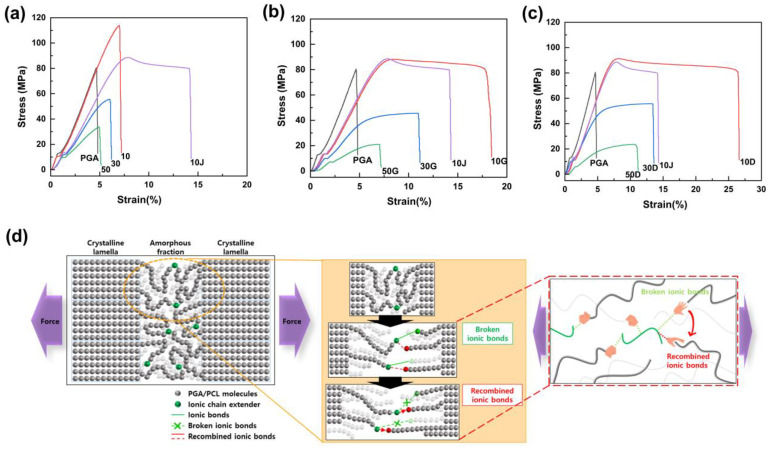
Stress–strain curves of PGA/PCL blending plastic sheets without CEs (**a**), with G-CE (**b**), and with D-CE (**c**). The schematic diagram of ionic bonding reaction (**d**).

**Figure 4:**
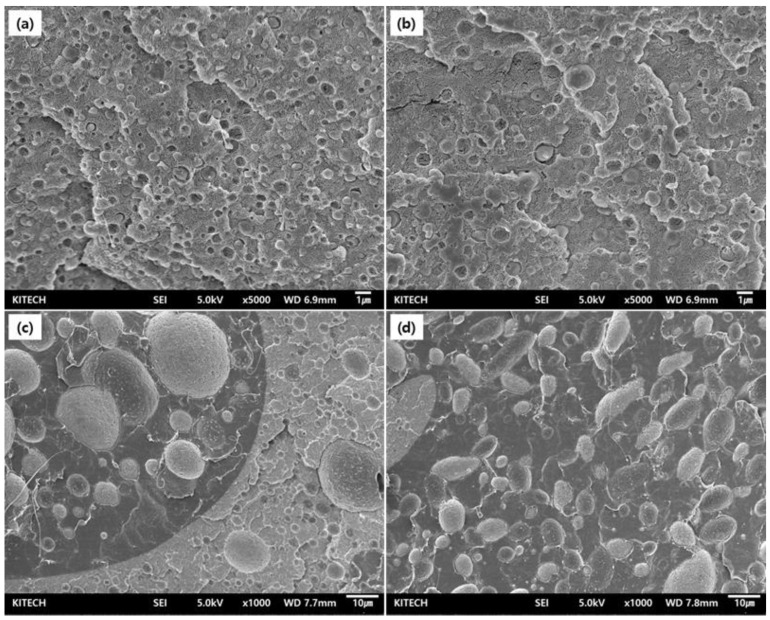
SEM images for a cross-section of PGA/PCL blending plastics with/without D-CE. PGA/PCL(90/10) without CE (**a**), PGA/PCL(90/10) with D-CE (**b**), PGA/PCL(50/50) without CE (**c**), and PGA/PCL(50/50) with D-CE (**d**).

Mechanical properties were measured, and ductility change behavior was evaluated, in order to confirm the effect of the chain extender using covalent or ionic bonds on the blending plastic matrix. Covalent G-type and ionic D-type chain extenders were analyzed by applying them to PGA/PCL blended plastics, each containing different PCL contents (Figure 3). In addition, the 10J in the graph is a PGA/PCL10 blending sample containing the Joncryl^®^ (ADR-4368), which is a typical chain extender used in the packaging industry, and it was prepared to compare the mechanical properties of the synthesized chain extenders. The elongation tended to decrease as the amount of PCL introduced increased with or without a chain extender, and it was confirmed that the same results of morphological behavior were shown in FE-SEM (Figure 3a and Figure 4). The compatibility was inhibited with increased content of PCL, and it can be inferred that this was due to the particle size of the dispersed PCL in the PGA matrix increasing and being more non-uniform.

Concurrently, the elongation rate increased and the tensile strength decreased according to the introduction of the two chain extenders, showing similar behavior (Figure 3b,c). However, the D-CE of ionic bonding showed increased ductility compared to the G-CE of the covalent bonding (Figure 3c). As the PCL content increased, the elongation rates when introducing G-CE were 18.5% at 10G, 11.2% at 30G, and 7.2% at 50G, respectively. And the samples introduced with D-CE were 26.6%, 13.60%, and 11.20% at 10D, 30D, and 50G, respectively. Compared to the case of PCL 10 wt% blending, which showed the most significant change in ductility, pure blending plastic without CE increased by about 3.69 times compared to 7.2% of blending plastic with ionic bond D-CE. In addition, all samples introduced with ionic-bonded D-CE showed relatively higher tensile strength than covalently bonded G-CE. The tensile strength of the blended plastic according to the type of chain extender was 88.2 MPa at 10G and 91.3 MPa at 10D, 46.5 MPa at 30G and 55.8 MPa at 30D, and 21.1 MPa at 50G and 23.6 MPa at 50D, respectively (Table S4).

Figure 3d’s schematic diagram shows that the ionic bond reacted in the amorphous region is pulled in both directions. The bond is broken and the recombination with a new adjacent carboxyl group is repeated. The quaternary ammonium can increase the fluidity of the polymer matrix and reduce frictional resistance under shear stress by causing a decrease in pour viscosity. It was argued that this was due to the repeated cleavage and recombination of ionic bonds. This study clearly showed this tendency, indicating a higher level of ductility than the existing covalent chain extenders [34].

### 3.5. Morphology Behavior of PGA/PCL Blending Polymers Matrix According to Type of Chain Extenders

The morphology behavior was analyzed to confirm the miscibility change between PGA and PCL by the ionic chain extender (Figure 4 and Figure S4) because mechanical properties are closely related to the shape and compatibility of the complex [37].

The cross-section of the PGA/PCL blending plastic, which blended 10 wt% of PCL without CE, showed a size of about 0.2–1 μm dispersed in the PGA matrix (Figure 4a). And the blending plastic with D-CE indicated that the size is uniformly distributed at about 0.5–0.7 μm (Figure 4b). This suggests that the condensation was relieved by the interaction with the PGA interface by the chain extender without the PCL particles independently aggregating. These properties are similar to existing covalent chain extenders. This shows that ionic chain extenders with relatively low bond strength can also play a sufficient role in improving the compatibility of PGA and PCL. In addition, this compatibility improvement is responsible for the increase in ductility described above.

In particular, PCL particles form non-uniform ‘islands’ of about 100 μm in size when PGA and PCL are blended at a ratio of 50 wt% without CE. Furthermore, the phase is partially reversed to form PGA in the PCL area and dispersed as particles (Figure 4c,d).

On the other hand, a phase inversion of some polymers was observed even when D-CE was added, but the size of the dispersed particles in the matrix was relatively uniform. The uniform dispersion of PGA and PCL can provide balanced strength. Moreover, increased ductility due to improved compatibility can be confirmed, as shown in the previous mechanical property analysis results [38].

## 4. Discussion

A novel ionic chain extender for controlling the ductility of PGA was studied. Among the amorphous blending polymers, PCL capable of ionic bonding was selected and blended to confirm the effect of the ionic chain extender. Through the MFI behavior, it was possible to establish the processing conditions of PGA within 6 min, and it was confirmed that the covalent (G-CE) and non-covalent (D-CE) bond chain extenders could lower the melt viscosity of the blending plastics. Exceptionally, the non-covalent ionic chain extender (D-CE) showed a sharp change in MFI as the introduced content increased, unlike the covalent chain extenders. This suggests that it may be more advantageous to control the melt viscosity.

Significantly increased ductility was confirmed in all cases where G-CE and D-CE were applied to PGA/PCL blending plastics, respectively. Compared to the elongation of 7.2% with PGA 90 wt% and PCL 10 wt% blending plastic without chain extender, the blending plastic with D-CE (10D) showed the most significant change in ductility with an elongation of 26.6%. Moreover, these ionic-bonded blending plastics with D-CE exhibited relatively higher tensile strengths than the covalent bonded blending plastics with G-CE. As the amount of PCL increased, the elongation increased, but the tensile strength decreasee, similar to the trade-off of elongation and tensile strength of available polymers.

This trend was also confirmed in the cross-sectional morphology of the blending plastics, showing that the PCL was distributed in the form of ‘islands’ as the content increased. The ‘islands’ dispersion hindered the interactions between the PGA matrix and PCL and lowered the dispersibility, reducing the tensile strength and elongation.

Despite this tendency, it was confirmed that the elongation and tensile strength increased with the ionic bonded chain extender (D-CE), compared to without chain extenders, with a high content of PCL. Each chain extender improved the compatibility between PGA and PCL, and the ductility of the PGA/PCL blending plastics was more greatly enhanced with non-covalently bonding D-CE than with covalently bonding G-CE.

Therefore, it was confirmed that the ionic bond proposed in this study could exert a sufficient effect as a chain extender, like a covalent bond.

## Figures and Tables

**Table 1:** Compositions of PGA/PCL blending plastics and PGA/PGA/CE blending plastic samples.

Sample Name	PGA (g)	PCL (g)	D-CE *^a^ (g)	G-CE *^b^ (g)	ADR-4368 *^c^ (g)
PGA	150	-	-	-	-
10	135	15	-	-	-
30	105	45	-	-	-
50	75	75	-	-	-
10D (10D_0.2)	135	15	0.30	-	-
10D_0.5	135	15	0.75	-	-
10D_1.0	135	15	1.50	-	-
30D	105	45	0.30	-	-
50D	75	75	0.30	-	-
10G_0.2	135	15	-	0.30	-
10G (10G_0.5)	135	15	-	0.75	-
10G_1.0	135	15	-	1.50	-
30G	105	45	-	0.30	-
50G	75	75	-	0.30	-
10J	135	15	-	-	1.50

*^a^: Ionic chain extender (D-CE). *^b^: Covalent chain extender (G-CE). *^c^: Joncryl^®^ chain extender purchased from BASF Co. Ltd.

## Data Availability

The data that support the findings of this study are available from the corresponding author upon reasonable request.

## Supplementary Materials

The following supporting information can be downloaded at: https://www.mdpi.com/article/10.3390/polym15143025/s1, Figure S1: Chemical structure analysis of synthesized covalent a) and non-covalent b) chain extenders. Table S1: Molecular weight analysis results of ionic bond chain extender (D-CE) and covalent bond chain extender (G-CE). Table S2: Melt flow index (MFI) results of PGA/PCL blending plastics according to blending ratio and introducing with covalent or non-covalent chain extenders. Figure S2. Thermogravimetric Analyzers (TGA) results of ionic bond chain extender (CE-D) and covalent bond chain extender (CE-G). Figure S3. Results of differential scanning calorimetry (DSC) analysis of PGA and PGA/PCL blended plastics (a) and enlarged data to confirm the T_g_ of blended plastics (b). Table S3: Differential scanning calorimetry (DSC) of PGA and PGA/PCL blending plastics with or without chain extenders. Table S4: Mechanical properties of PGA/PCL blending plastics according to blending ratio and introducing with or without chain extenders. Figure S4. SEM images for cross-section of PGA (a) and PGA/PCL blending plastics with/without D-CE (b–i).
