# Supplementary material for: Ductile Effect of PGA/PCL Blending Plastics Using a Novel Ionic Chain Extender with Non-Covalent Bonds
Source: Polymers (Basel). 2023 Jul 12;15(14):3025. doi: 10.3390/polym15143025 (PMC10385193; doi:10.3390/polym15143025)
Supplement: Supplementary file 1 [file polymers-15-03025-s001.zip › polymers-2462625-supplementary.pdf]

Supporting information

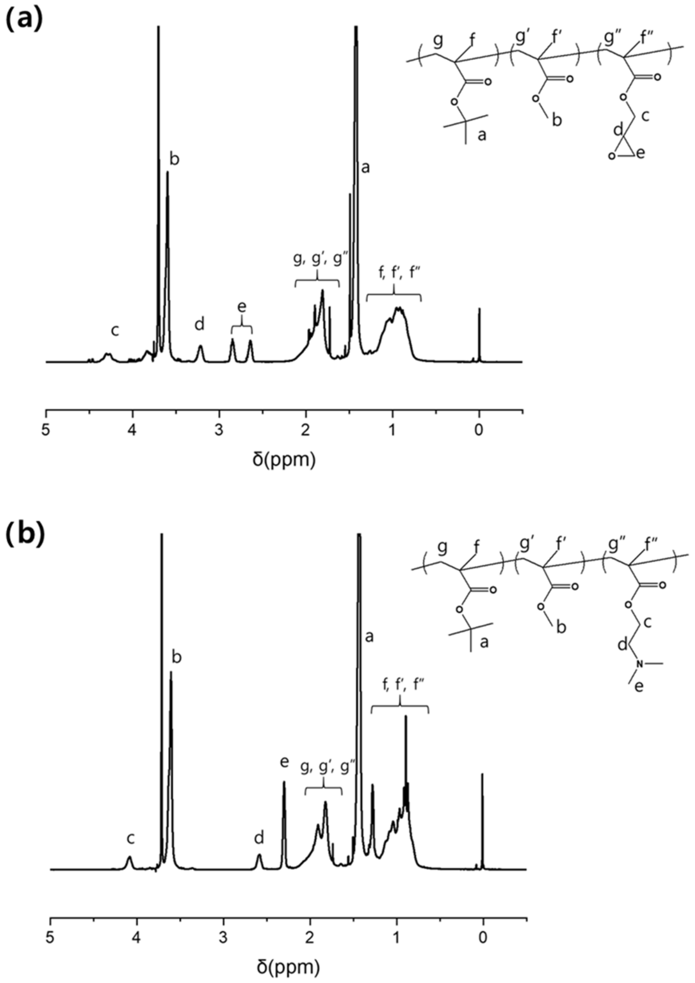

Figure S1. Chemical structure analysis of synthesized covalent (a) and non-covalent (b) chain extenders. The segment ratio of the synthesized covalent chain extender (G-CE) is GMA/ter-BMA/MMA = 18.6/38.9/42.5, and the segment ratio of the synthesized ionic chain extender (D-CE) is DMAEMA/ter-BMA/MMA = 8.8/42.0/49.2.

Table S1. Molecular weight analysis results of ionic bond chain extender (D-CE) and covalent bond chain extender (G-CE).

Sample name	Mn (g/mol)	Mw (g/mol)	Mp (g/mol)	Mw/Mn (arb. units.)
G-CE*[a]	30,300	143,000	92,600	4.73
D-CE*[b]	7,040	47,200	58,100	6,70

*[a] ; Ionic chain extender (D-CE)

*[b] ; Covalent chain extender (G-CE)

Table S2. Melt flow index (MFI) results of PGA/PCL blending plastics according to blending ratio and introducing with covalent or non-covalent chain extenders.

Sample name	PGA (g)	PCL (g)	G-CE*[a] (g)	D-CE*[b] (g)	ADR-4368*[c] (g)	Time (min)	MFI (g/10min)
PGA_0	150	-	-	-	-	0	24.5
PGA_4	150	-	-	-	-	4	25.9
PGA	150	-	-	-	-	6	26.9
PGA_8	150	-	-	-	-	8	30.6
PGA_10	150	-	-	-	-	10	31.5
PGA_12	150	-	-	-	-	12	32.9
10	135	15	-	-	-	6	37.0
10G	135	15	0.75	-	-	6	15.6
10D	135	15	-	0.3	-	6	22.7
10GD	135	15	0.75	0.3	-	6	21.1
10J	135	15	-	-	1.5	6	38.9
30	105	45	-	-	-	6	63.4
30G	105	45	0.75	-	-	6	18.3
30D	105	45	-	0.3	-	6	24.7
30GD	105	45	0.75	0.3	-	6	24.8
50	75	75	-	-	-	6	44.1
50G	75	75	0.75	-	-	6	17.9
50D	75	75	-	0.3	-	6	32.4
50GD	75	75	0.75	0.3	-	6	27.8
10G_0.2	135	15	0.3	-	-	6	18.4
10G_0.5 (10G)	135	15	0.75	-	-	6	15.6
10G_1.0	135	15	1.5	-	-	6	16.3
10D_0.2	135	15	-	0.3	-	6	22.7
10D_0.5 (10D)	135	15	-	0.75	-	6	44.7
10D_1.0	135	15	-	1.5	-	6	52.8

*[a] ; Ionic chain extender (D-CE)
*[b] ; Covalent chain extender (G-CE)
*[c] ; Joncryl® chain extender purchased from BASF Co. Ltd

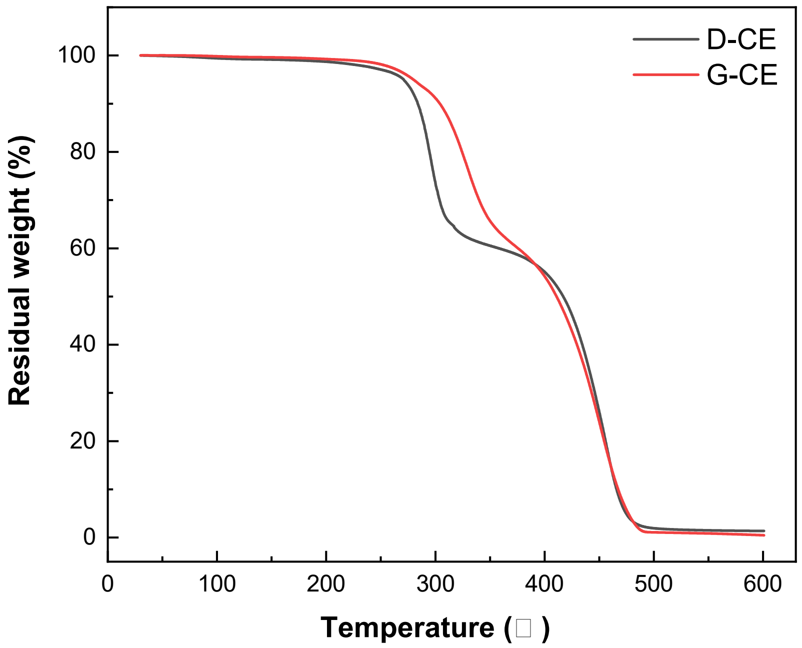

Figure S2. Thermogravimetric analysis (TGA) results of ionic bond chain extender (D-CE) and covalent bond chain extender (G-CE).

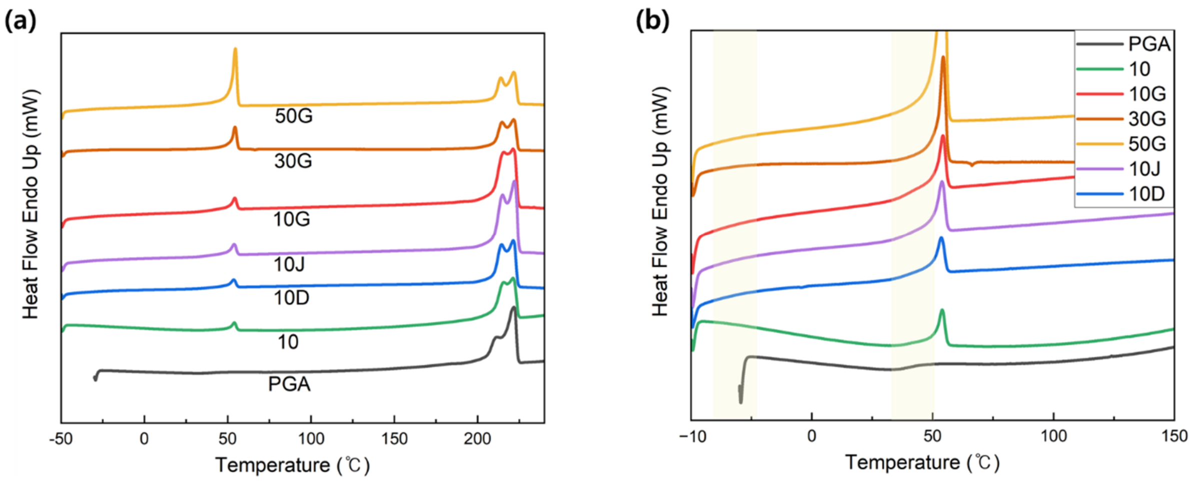

Figure S3. Results of differential scanning calorimetry (DSC) analysis of PGA and PGA/PCL blended plastics (a) and enlarged data to confirm the T_g of blended plastics (b).

Table S3. Differential scanning calorimetry (DSC) of PGA and PGA/PCL blending plastics with or without chain extenders.

Sample name	$T_{c1\ at\ PCL}$ (℃)	area (mJ)	$T_{c2\ at\ PGA}$ (℃)	area (mJ)	$T_{m1\ at\ PGA}$ (℃)	$T_{m2\ at\ PGA}$ (℃)
PGA	-	-	193.5	536.2	210.8	221.7
10	13.8	16.5	193.1	281.2	214.8	222.2
10G	14.7	32.3	190.9	389.9	215.2	221.3
10D	11.6	19.1	192.4	283.3	214.0	221.3
10GD	11.4	10.1	193.1	172.9	215.6	222.6

Table S4. Mechanical properties of PGA/PCL blending plastics according to blending ratio and introducing with or without chain extenders.

Sample name	Tensile strength (MPa)	Elongation (%)
PGA	80.6	4.8
10	113.8	7.2
30	55.6	6.2
50	33.8	5.2
10G	88.2	18.5
30G	46.5	11.5
50G	21.1	7.2
10D	91.3	26.6
30D	55.8	13.6
50D	23.6	11.2
10J	88.6	14.4

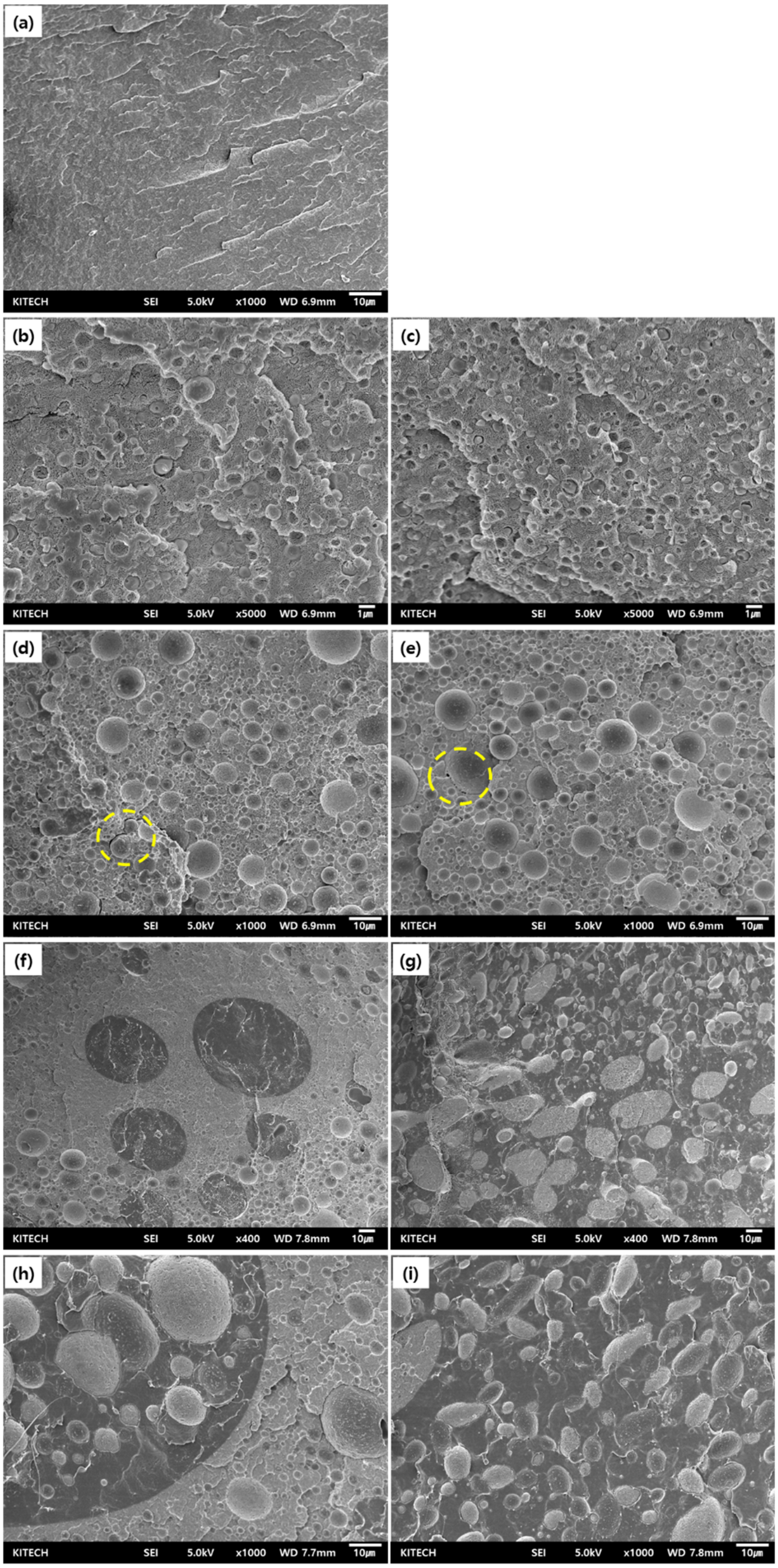

Figure S4. SEM images for cross-section of PGA (a) and PGA/PCL blending plastics with/without D-CE (b-i). PGA/PCL(90/10) without CE (b) and with D-CE (c), PGA/PCL(70/30) without CE (d) and

with D-CE (e), 400 magnification of PGA/PCL(50/50) without CE (f) and with D-CE (e), 1,000 magnification of PGA/PCL(50/50) without CE (h) and with D-CE (i).
